# Results of Eustachian tube balloon dilation measured using the nine-step test

**DOI:** 10.1038/s41598-023-44812-1

**Published:** 2023-10-18

**Authors:** Seong Hoon Bae, Seungmin Kwak, Ji Hyuk Han, Jinsei Jung, Sung Huhn Kim, Jae Young Choi, In Seok Moon

**Affiliations:** 1 Department of Otorhinolaryngology, Gangnam Severance Hospital, Yonsei University College of Medicine, 211 Eonju-ro, Seoul, 06229, Republic of Korea; 2 Department of Otorhinolaryngology, Severance Hospital, Yonsei University College of Medicine, 50-1 Yonsei-ro, Seodaemun-gu, Seoul, 03722 Republic of Korea

**Keywords:** Diseases, Medical research, Outcomes research

## Abstract

Suggested several decades ago, the nine-step test is an intuitive test of Eustachian tube function. However, studies employing the nine-step test to assess the results of Eustachian tube balloon dilation (EBD) are limited. We aimed to objectively evaluate the efficacy of EBD in opening failure patients with decreased maximal peak pressure difference (MPD) using the nine-step test. Patients who had MPD values ≤ 13 daPa in the nine-step test were enrolled. The patients were categorized into two groups according to treatment decisions after discussion with a clinician: an EBD group (N = 26) and a medication group (N = 30). One month after treatment, the seven-item Eustachian Tube Dysfunction Questionnaire (ETDQ7) and the nine-step test were administered to all participants and subgroups of symptomatic participants (ETDQ7 > 15). MPD improved (increased) in both the EBD group and the medication group. ETDQ7 values improved (decreased) in the EBD group, but not in the medication group. In subgroup analysis, MPD and ETDQ7 values improved only in the symptomatic EBD group. According to the nine-step test, EBD can normalize 53.8% of decreased MPD. Posttreatment MPD and ETDQ7 scores were significantly better in the EBD group than in the medication group. However, EBD in patients with abnormal nine-step test results seemed less efficacious when the treatment results of the medication group were considered.

## Introduction

Eustachian tube dysfunction (ETD) is characteristic of several otologic diseases and symptoms^1,2^. Although the diagnostic criteria for ETD are not firmly defined, there have been efforts to reach a consensus in Europe and America^3,4^. Similarly, the indications and contraindications for Eustachian tube balloon dilation (EBD) also differ from study to study. However, reports of promising results with EBD are growing in number^5^.

Given that classical treatment of ETD is confined to medication, topical drug administration, and tympanostomy tube, EBD offers an advantage in terms of the durability of symptom control^6^. Moreover, compared surgical management, EBD is less invasive and offers better symptom control^7,8^. Furthermore, a meta-analysis study in terms of subjective symptom improvement, as > 90% of patients treated with EBD showed decreased Seven-item Eustachian Tube Dysfunction Questionnaire (ETDQ7) scores^9^. However, objective results from tympanometry or Valsalva maneuver tests have raised doubts about the effectiveness of EBD in terms of normalization rates^9^.

Notwithstanding, there is no gold standard test for objectively evaluating the function of the Eustachian tube. The nine-step test is a classical Eustachian tube function test introduced by Bluestone et al. several decades ago^10^. It reflects the physiological opening of the Eustachian tube during swallowing. However, a drawback of this test is that the tympanic membrane should be intact and show a distinct peak in the tympanometry test. To date, reports of EBD results measured using the nine-step test are limited, although the nine-step test is a physiologic and intuitive test for evaluating physiologic opening of the Eustachian tube. Additionally, a report on EBD results in abnormal nine-step test patients is also lacking.

Therefore, in this study, we assessed nine-step test results before and after EBD in opening failure patients with decreased middle ear pressure (MEP) difference post-swallowing during inflation/deflation (maximal peak-pressure difference, MPD). As a control, a medication group, which showed similar results in the nine-step test but decided on medical treatment rather than EBD, was enrolled. Treatment results, including ETDQ7, MEP, and nine-step test results, were comparatively analyzed between the two groups. Subgroup analysis among symptomatic participants (ETDQ7 > 15) was additionally performed. We expect that the results from the nine-step test will provide objective evidence on the clinical effects of EBD and shed light on the efficacy of EBD in patients with abnormal nine-step test results.

## Results

### Participants

In the EBD group, 47 ears that underwent EBD without any other procedures (e.g. ventilation tube insertion) were screened. Overall, 26 ears were enrolled in the EBD group after excluding 21 ears fulfilling the exclusion criteria. In the medication group, 62 ears were screened, and 32 ears were excluded in accordance with the exclusion criteria. Ultimately, 26 and 30 ears were included in the EBD and medication groups, respectively. There were two missing values (omitted pretreatment survey) for ETDQ7 in each group. Demographic data did not differ between the two groups (Table 1). In preoperative evaluation, ETDQ7 was significantly better in the medication group (*P* = 0.044). However, objective results from the nine-step test were not different.

**Table 1:** Participant pre-treatment information.

	EBD group	Medication group	*P*-value
Median age, years (IQR)	41.5 (31.25)	53.0 (33.5)	0.072
Male sex, N (%)	9 (34.6)	10 (33.3)	1.000
Right side, N (%)	12 (46.2)	19 (63.3)	0.282
Tympanometry, N (%)			0.785
Type A	17 (65.4)	18 (60.0)	
Type C	9 (34.6)	12 (40.0)	
Median MPD, daPa (IQR)	5.0 (4.25)	4.5 (5.5)	0.993
Median MEP, daPa (IQR)	− 15 (76.5)	− 20.5 (78.0)	0.730
Median ETDQ7 (IQR)	23.5 (16.25)^a^	15.0 (12.25)^b^	0.044*
Participants, N (%)	26 (100)	30 (100)	

EBD, Eustachian tube balloon dilation; IQR, interquartile range; N, number; MPD, maximal peak-pressure difference; daPa, decapascals; MEP, middle ear pressure; ETDQ7: Eustachian tube dysfunction questionnaire.

^a^Two missing values in the EBD group.

^b^Two missing values in the medication group.

*P < 0.05.

### Treatment outcomes

One month after treatment, the maximal peak pressure difference (MPD, *P* = 0.016) and ETDQ7 (*P* = 0.046) showed better results in the EBD group than the medication group (Table 2). However, MEP and tympanometry results did not differ between the groups. The cure rate of MPD (normalized to > 13 daPa) was higher in the EBD group (53.8% vs. 30.0%, *P* = 0.103); however, the difference was not statistically significant.

**Table 2:** Follow-up evaluation at 1 month after treatment for all participants.

	EBD group	Medication group	*P*-value
Tympanometry, N (%)			0.755
Type A	21 (80.8)	23 (76.7)	
Type C	5 (19.2)	7 (23.3)	
Median MPD, daPa (IQR)	15.0 (30.25)	8.0 (16.5)	0.016*
Median MEP, daPa (IQR)	− 8.0 (32.25)	− 20.0 (48.25)	0.565
Median ETDQ7 (IQR)	11.5 (8.75)^a^	19.0 (16.75)	0.044*
Cure in MPD, N (%)	14 (53.8)	9 (30.0)	0.103
Participants, N (%)	26 (100)	30 (100)	

EBD, Eustachian tube balloon dilation; IQR, interquartile range; N, number; MPD, maximal peak pressure difference; daPa, decapascals; MEP, middle ear pressure; ETDQ7: Eustachian tube dysfunction questionnaire.

^a^Two missing values in EBD group.

^b^One missing values in medication group.

*P < 0.05.

**Figure 1:**
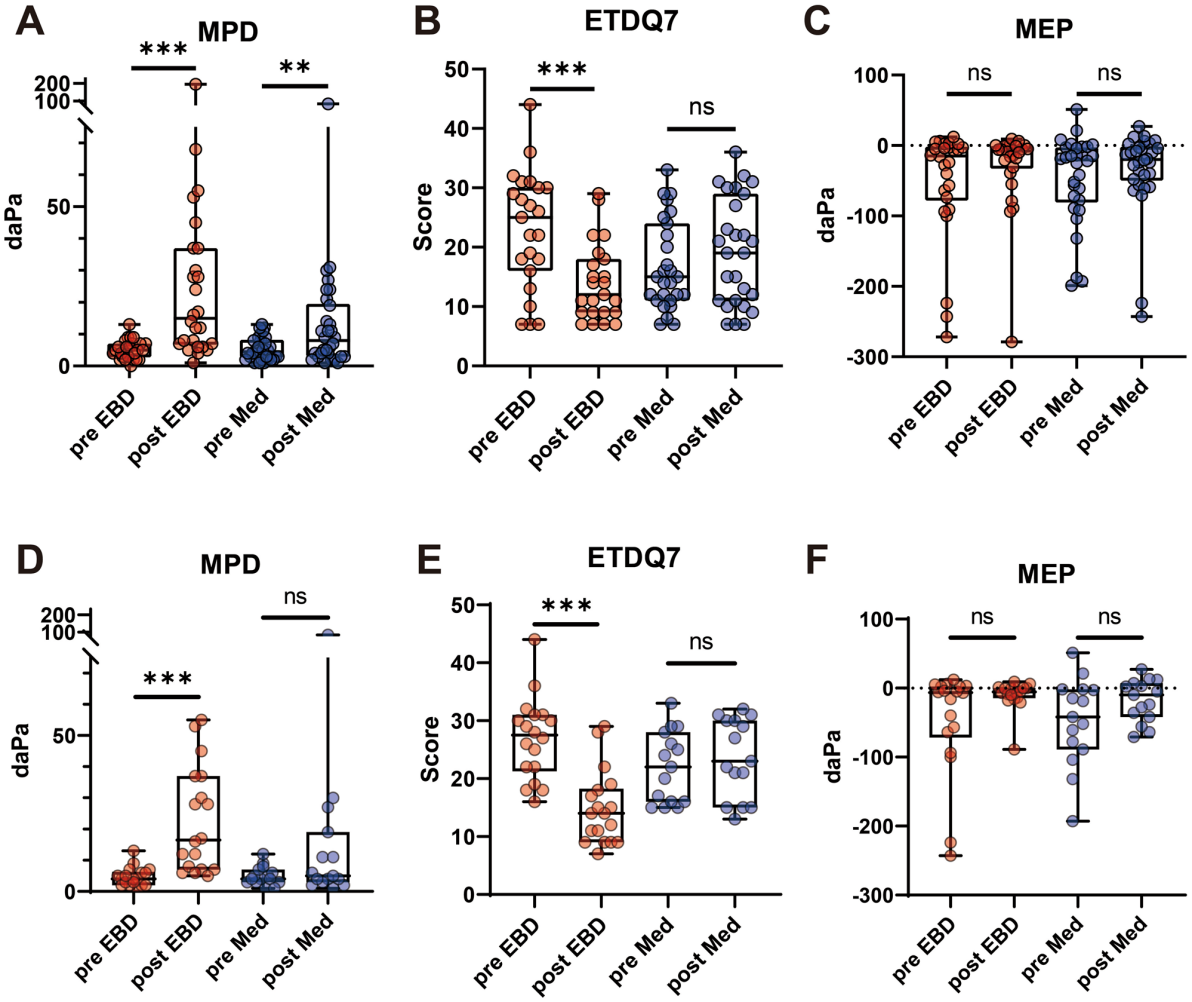
Treatment outcomes for the EBD and medication groups at 1-month follow-up evaluation. Maximal peak-pressure difference for all participants (**A**) and symptomatic participants (**D**). Eustachian tube dysfunction questionnaire scores for all participants (**B**) and symptomatic participants (**E**). Middle ear pressure for all participants (**C**) and symptomatic participants (**F**). The floating box and midline indicate interquartile range and median values, respectively. Whiskers indicate the minimum to maximum range. Each circle indicates an individual result. EBD, Eustachian tube balloon dilation; Med, medication; MPD, maximal peak-pressure difference; daPa, decapascal; MEP, middle ear pressure; ETDQ7, Eustachian tube dysfunction questionnaire, ns, not significant; **P < 0.01, ***P < 0.001.

To analyze treatment efficacy in both groups, a paired match analysis was conducted for before and after treatment results (Fig. 1A–C). After treatment, both groups showed significant improvements in MPD (*P* < 0.001 and *P* = 0.008 in the EBD and medication groups, respectively). Regarding ETDQ7, only the EBD group showed a significant improvement (*P* < 0.001 and *P* = 0.122 in the EBD and medication groups, respectively). Also, neither group showed a significant difference in MEP (*P* = 0.309 and *P* = 0.373 in the EBD and medication groups, respectively).

In subgroup analysis with symptomatic participants, results were similar, although significant MPD improvement (*P* < 0.001 and *P* = 0.197 in the EBD and medication groups, respectively) was identified only in the EBD group (Fig. 1D–F). The cure rate of MPD was similar to that for all participants, 55.6% and 26.7% in the EBD and the medication group, respectively (Table 3). ETDQ7 was also much improved in the EBD group after treatment, although there was a significant difference in preoperative ETDQ7.

**Table 3:** Outcomes for symptomatic participants.

	EBD group	Medication group	*P*-value
Median age, years (IQR)	52.0 (32.5)	56.0 (39.0)	0.361
Male sex, N (%)	5 (27.8)	5 (33.3)	1.000
Right side, N (%)	8 (44.4)	9 (60.0)	0.491
Tympanometry pre-op, N (%)			0.493
Type A	12 (66.7)	8 (53.3)	
Type C	6 (33.3)	7 (46.7)	
Tympanometry post-op, N (%)			0.308
Type A	17 (94.4)	12 (80.0)	
Type C	1 (5.6)	3 (20.0)	
Median MPD, daPa (IQR)			
Pre-op	4.0 (4.25)	4.0 (4.0)	0.735
Post-op	16.5 (30.0)	5.0 (16.0)	0.011*
Median MEP, daPa (IQR)			
Pre-op	− 6.5 (73.75)	− 42.0 (87.0)	0.556
Post-op	− 5.5 (14.5)	− 10.0 (49.0)	0.735
Median ETDQ7 (IQR)			
Pre-op	27.5 (9.75)	22.0 (12.0)	0.036*
Post-op	14.0 (9.25)	23.0 (15.0)	0.001*
Cure in MPD, N (%)	10 (55.6)	4 (26.7)	0.158
Cure in ETDQ7, N (%)	10 (55.6)	1 (6.7)	0.004*
Participants, N (%)	18 (100)	15 (100)	

EBD, Eustachian tube balloon dilation; IQR, interquartile range; N, number; MPD, maximal peak pressure difference; daPa, decapascals; MEP, middle ear pressure; ETDQ7: Eustachian tube dysfunction questionnaire.

*P < 0.05.

### Sequential results of medication and EBD

**Figure 2:**
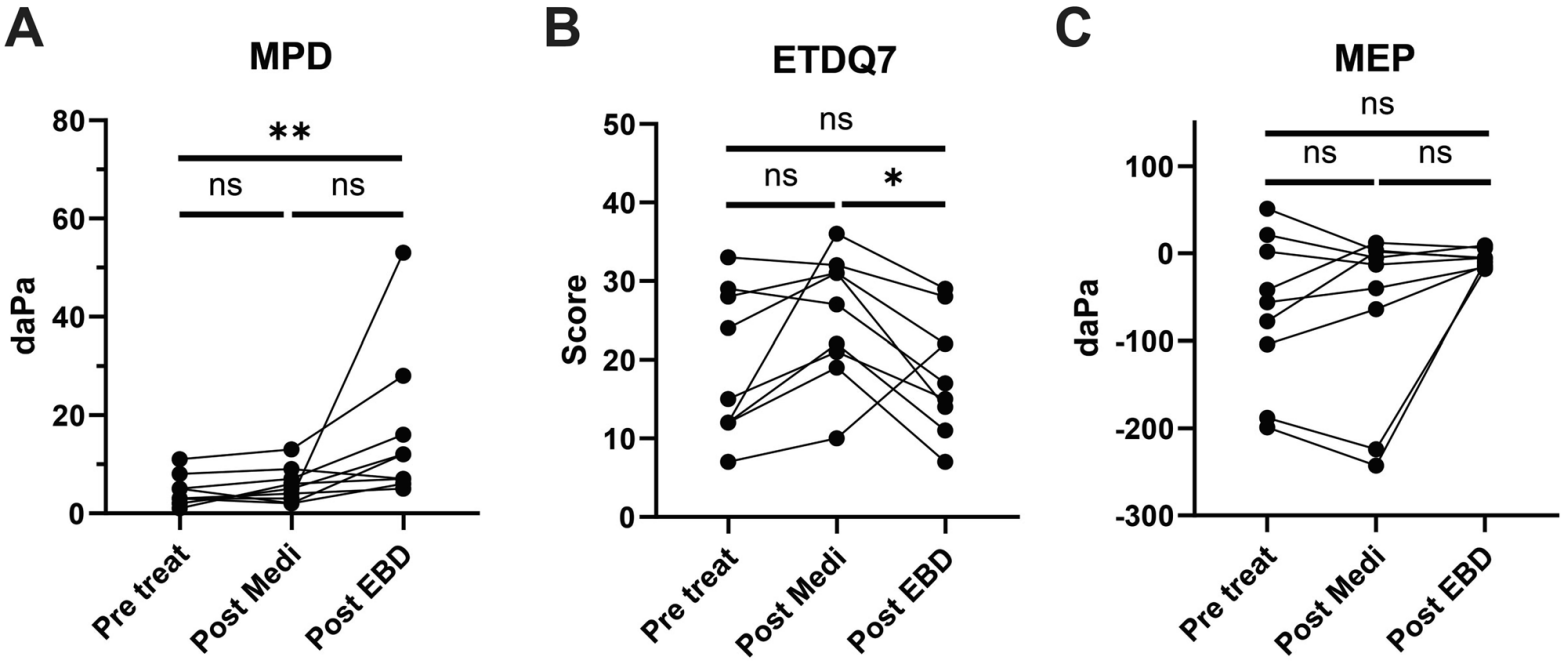
Treatment outcomes of the sequentially (medication followed by EBD) treated patients. (**A**) Maximal peak-pressure difference. (**B**) Eustachian tube dysfunction questionnaire score. (**C**) Middle ear pressure. Each connected line and circle indicate an individual result. EBD, Eustachian tube balloon dilation; Med, medication; MPD, maximal peak-pressure difference; daPa, decapascal, MEP, middle ear pressure; ETDQ7, Eustachian tube dysfunction questionnaire; ns, not significant; *P < 0.05; **P < 0.01.

Nine ears were sequentially treated with medication, followed by EBD because their symptoms did not improve after medical therapy. The washout period between the end of medication and EBD was 1 month. The cure rate of EBD in these medication-refractory ears was 33.3% (3 ears), and that of ETDQ7 was also 33.3% (3 ears). Tympanometry type C was recorded in 55.6% (5 ears) before treatment, 33.3% (3 ears) after medication, and 0% after EBD. In pairwise statistical analysis, MPD between pre-treatment and EBD (*P* = 0.010) showed significant improvement (Fig. 2). ETDQ7 scores between the medication and EBD groups (*P* = 0.020) also showed a significant improvement; however, other comparisons, including MEP, were not significant.

## Discussion

First of all, to prevent misunderstanding, we should note that the participants enrolled in this study are not classical ETD patients; they are abnormal nine-step test subjects with or without ETD symptoms. We chose this population to investigate the efficacy of EBD, as reflected objectively in the nine-step test, in terms of whether EBD can cure opening failure of the Eustachian tube. In this study, the treatment efficacy of EBD was superior to that of medication. It is not novel to suggest that EBD is effective; however, we were able to substantiate available evidence using another measurement modality. In addition, the control group taking medication showed partial improvement, which is inconsistent with previous studies^11,12^. Our study supports the use of the nine-step test as an alternative modality for objectively evaluating the treatment results of EBD and indicates an abnormal nine-step test result as a novel indication for EBD.

The efficacy of EBD has been demonstrated using several methods. The most popular modality appears to be normalization of tympanometry. There are several representative randomized control trials using tympanometry to evaluate the results of EBD^11–14^. Anand et al. reported that MEP was normalized in 51% of patients after 6 weeks of EBD^11^. Liang et al. reported that tympanometry type changed from type B to type A in 23.3% after 1 month of EBD^13^. A study using the Eustachian Tube Score system reported a normalization rate of 70% after 3 months of EBD^15^. All studies showed a significantly better objective outcome with EBD, compared to controls, in short-term follow-up. Our study also showed significantly better outcomes in several values post-treatment; however, the cure rate of MPD was not significantly different because the medication group also showed significant improvement (*P* = 0.008) after treatment. In addition, MEP was not different between EBD and medication groups. This may be related to defining MPD ≤ 13 daPa as an abnormal nine-step test result rather than classical ETD patients: for instance, this study included patients with MPD ≤ 13 daPa even for tympanometry type A or ETDQ7 < 15. Furthermore, patients with type B tympanometry were excluded because the nine-step test is not applicable to them. The low efficacy of EBD (53.8% cure rate in MPD) in this study seems to have resulted from the different patient selection and a different test modality. Given that the nine-step test applies external auditory canal air pressure, it is likely to mimic baro-challenge-induced Eustachian tube dysfunction^16,17^. Taken together, the appropriate selection of patients for EBD may be an important issue that should be further investigated in the future.

Initially, ETDQ7 scores differed between the EBD and medication groups, possibly because patients with more severe subjective symptoms tended to choose surgical treatment. Changes in ETDQ7 scores after treatment were significant only in the EBD group. However, this was not a blinded study, and the placebo effect was not excluded. To reduce selection bias, the cure rate of ETDQ7 was analyzed from subgroups of symptomatic patients whose ETDQ7 scores were higher than 14 before treatment. In this subgroup analysis, a significantly better cure rate of ETDQ7 in the EBD group (55.6% vs. 6.7%, *P* = 0.004) was found, although small numbers of patients were included.

In terms of objective results, MEP did not differ between the groups. However, MPD was significantly higher (*P* = 0.016) in the EBD group after 1 month of treatment, although it did not differ initially between the groups. Higher MPD in the EBD group was also identified in subgroup analysis (*P* = 0.011). Sequentially treated patients showed significantly better outcomes in comparison of pre-treatment and post-EBD status (*P* = 0.010). Conclusively, EBD was effective in both the objective nine-step test and the subjective ETDQ7 in patients with abnormal nine-step test results initially.

This study has some limitations that warrant consideration. Most of them are due to the retrospective study design, demanding randomized prospective studies in the future. One limitation is the lack of long-term follow-up. Although studies have shown that the effects of EBD appear within 1 month of treatment^12,18^, some studies have shown that normalization of the retracted tympanic membrane is slow^11,19^. Given the long-term results of previous studies, the EBD group could show better outcomes if they were observed over a longer period than 1 month. Also, there could be selection bias because the treatment was not randomly decided. Indeed, younger age (although not significant) and severe symptoms (higher ETDQ7) were identified in the EBD group. However, we do not believe that this difference would affect the objective results (MPD and MEP) measured by the nine-step test because the objective results were similar between groups in pre-op evaluation. Another limitation is that the enrolled patients in this study are not classical ETD patients, as mentioned above. For instance, tympanometry type B ears and ears with a severely adhesive drum, which were not clearly tympanometry type A or C, were excluded. Further, some of the enrolled ears were asymptomatic (ETDQ7 < 15) before 1 month from the day of visiting clinic; this is because the purpose of this study was to objectively validate EBD efficacy in the normalization of the opening failure of the Eustachian tube, not primarily focused on validating the treatment efficacy of ETD symptoms. Also, possible confounding factors (e.g., pharyngeal video endoscopy, allergic rhinitis, and gastric acid reflux) that should be properly controlled were omitted in the study^20–22^. Finally, this study did not include a non-treatment control group, which may better indicate the efficacy of EBD.

In conclusion, EBD can normalize 53.8% of opening failures observed in the nine-step test. Post-treatment MPD and ETDQ7 scores were significantly better in the EBD group than in the medication group. However, the efficacy of EBD in patients with abnormal nine-step test results was not very high when considering the treatment results of the medication group.

## Methods

### Study design and setting

This study was conducted as a single-center retrospective case–control study at a tertiary referral university hospital (Severance hospital, Seoul, Korea) from January 6, 2021 to January 3, 2023. Patients with ear fullness who visited our hospital routinely underwent the nine-step test and ETDQ7 after otoscopic inspection to exclude patulous eustachian tube or otitis media. With each patient, clinicians suggested two potential treatment options, medication and EBD, after discussing their symptoms and test results if ear fullness had lasted longer than 3 months. Because ETDQ7 asks about symptoms within 1 month, patients whose ETDQ7 < 15 were also enrolled for treatment when they had intermittent symptoms and wanted to be treated (including baro-challenge-induce ETD, recurrent otitis media, etc.). EBD was performed using a Navilloon-e device (Mega Medical, Seoul, Korea), according to the manufacturer’s instructions (2 min, 12 bar). No medications were prescribed after EBD during the follow-up period. For medical treatment without EBD, fluticasone nasal spray (Avamys nasal spray; GlaxoSmithKline, London, UK) and pseudoephedrine hydrochloride (Actifed; Samil Pharmaceutical, Seoul, Korea) were prescribed for 1 month. A follow-up nine-step test and ETDQ7 were scheduled for 1 month after EBD or at the completion of medication. Patient data were obtained from the Severance Hospital database.

The study protocol was approved by the institutional review board of Severance Hospital (Project, 4-2023-0063). The requirement for informed consent was waived due to the retrospective nature of this study. This manuscript followed the STROBE Reporting Guidelines for Case–control Studies. This study was performed according to the approved protocol and the guidelines of the Declaration of Helsinki.

### Participants

In this study, there were two groups: the EBD group and medication group. Patients who underwent EBD without any other procedures were included in the EBD group. The inclusion criterion of the medication group was completion of 1 month of medication for ETD. The exclusion criteria were as follows: (1) no distinguishable peak in tympanometry (not type A or C), (2) MPD in the nine-step test higher than 13 daPa, (3) lack of 1-month follow-up evaluation with the nine-step test, and (4) craniofacial anomaly. We selected patients with abnormal MPD (≤ 13 daPa) in whom opening of the Eustachian tube failed upon swallowing^23^.

### Eustachian tube function evaluation

All enrolled patients were asked to complete the Korean version of the ETDQ-7. The survey consisted of seven questionnaires regarding representative symptoms of ETD^24^. The participants also underwent otoscopic examination immediately followed by a Eustachian tube function test using the GSI TympStar Pro analyzer (Grason-Stadler Inc., Eden Prairie, MN, USA). The Eustachian tube function test was based on Bluestone’s nine-step test. Evaluation of middle ear pressure was conducted in a normal state, followed by the introduction of 400 daPa of negative pressure into the ear canal. The participants were asked to dry swallow three times, after which ear canal pressure returned to ambient pressure. Then, middle ear pressure was checked (middle ear pressure should be increased relative to the first middle ear pressure at adequate opening of Eustachian tube). The same procedure was performed using 400 daPa of positive pressure.

The maximal difference in middle ear pressure among the three states was defined as MPD. MEP was defined as middle ear pressure at rest. Tympanometry type A was defined as MEP > − 50 daPa, and type C was defined as MEP ≤ − 50 daPa^25^.

### Statistical methods

As continuous variables did not pass the normality test, nonparametric tests were used. The Mann–Whitney U test was used to compare two groups. The Wilcoxon matched-pairs signed-rank test was used to analyze variables before and after treatment. To analyze three states (before treatment, medication, and EBD), the Friedman test with Dunn’s multiple comparison test for post-hoc analysis was used. For proportional values, Fisher’s exact test (two-tailed) was used to evaluate statistical significance. According to a previous study^14^, 23 patients in each group are required to achieve an alpha error of 5% and a beta error of 20% at a power of 80%. Results were visualized using Prism 8.0 (GraphPad Software, San Diego, CA, USA). All statistical analyses were conducted using IBM SPSS version 20 (IBM Co., Armonk, NY, USA), and *P* < 0.05 was considered statistically significant.

## Data Availability

The data are available from the corresponding author upon reasonable request.
